# Melatonin Alleviates High Temperature-Induced Pollen Abortion in *Solanum lycopersicum*

**DOI:** 10.3390/molecules23020386

**Published:** 2018-02-11

**Authors:** Zhen-Yu Qi, Kai-Xin Wang, Meng-Yu Yan, Mukesh Kumar Kanwar, Dao-Yi Li, Leonard Wijaya, Mohammed Nasser Alyemeni, Parvaiz Ahmad, Jie Zhou

**Affiliations:** 1 Department of Horticulture/Zhejiang Provincial Key Laboratory of Horticultural Plant Integrative Biology, Zhejiang University, Hangzhou 310058, China; qizhenyu@zju.edu.cn (Z.-Y.Q.); keisha@zju.edu.cn (K.-X.W.); 11416057@zju.edu.cn (M.-Y.Y.); kanwar@zju.edu.cn (M.K.K.); 2 Agricultural Experiment Station, Zhejiang University, Hangzhou 310058, China; 3 Chinese Academy of Agricultural Mechanization Sciences, Beijing 10083, China; daoyili@126.com; 4 Department of Botany and Microbiology, Faculty of Science, King Saud University, Riyadh 11451, Saudi Arabia; lwijaya@ksu.edu.sa (L.W.); mnyemeni@ksu.edu.sa (M.N.A.); pganaie@ksu.edu.sa (P.A.)

**Keywords:** autophagy, heat shock protein (HSP), high temperature, melatonin, pollen, *Solanum lycopersicum*

## Abstract

Melatonin is a pleiotropic signal molecule that plays critical roles in regulating plant growth and development, as well as providing physiological protections against various environmental stresses. Nonetheless, the mechanisms for melatonin-mediated pollen thermotolerance remain largely unknown. In this study, we report that irrigation treatment with melatonin (20 µM) effectively ameliorated high temperature-induced inactivation of pollen and inhibition of pollen germination in tomato (*Solanum lycopersicum*) plants. Melatonin alleviated reactive oxygen species production in tomato anthers under high temperature by the up-regulation of the transcription and activities of several antioxidant enzymes. Transmission electron micrograph results showed that high temperature-induced pollen abortion is associated with a premature degeneration of the tapetum cells and the formation of defective pollen grains with degenerated nuclei at the early uninuclear microspore stage, whilst melatonin protected degradation of organelles by enhancing the expression of heat shock protein genes to refold unfolded proteins and the expression of autophagy-related genes and formation of autophagosomes to degrade denatured proteins. These findings suggest a novel function of melatonin to protect pollen activity under high temperature and support the potential effects of melatonin on reproductive development of plants.

## 1. Introduction

As the world warms up, plants are more vulnerable to extreme climates. High temperature impacts plant vegetative growth and reproductive development, and especially inhibits flower bud differentiation, the development of female and male gametophytes, blooming and fruit set [1,2]. The decline in yield of numerous crops with high temperature is mainly associated with pollen infertility and male sterility [3,4]. The earliest high temperature-induced meiotic abnormalities occur in the pollen development [5,6]. Chromosome behavior and meiotic cell division is affected, which leads to unbalanced chromosome separation between spores and formation of *2n* pollens after heat stress [5,7]. Heat stress also reduces pollen number and viability, which are supported by tapetum cells [8]. The development and degeneration processes of tapetum cells are disturbed and reactive oxygen species (ROS) are accumulated under extreme heat or long-term mild heat stresses [9]. In addition, high temperatures induce the abnormalities of microtubules and the location and distribution during pollen tube growth, which is more sensitive to increasing temperature than vegetative cells [10,11].

In vegetative stages, plants have a sophisticated thermotolerance network to maintain cellular homeostasis, including increasing membrane fluidity, enhancing specific enzyme activities, scavenging heat-induced ROS, and refolding or degrading denatured proteins [12,13,14,15]. However, little is known about these mechanisms in the pollen development process. High temperature-induced generation of sterile pollen due to higher accumulation of ROS, such as hydroxyl radicals (•OH), superoxide anion (O_2_•^−^) and hydrogen peroxide (H_2_O_2_), which induce lipid peroxidation and oxidation of nucleic acids, sugars and proteins, ultimately results in pollen and anther cell death [8,16]. Antioxidant system is activated to scavenge ROS and protect anthers upon exposure to high temperatures. The transcript level of *APX3* gene which encodes ROS-scavenging enzyme is up-regulated to reduce ROS accumulation in tomato (*Solanum lycopersicum*) microspores [17]. In contrast, dysfunction of an antioxidant system in the male reproductive organs leads to a continuous accumulation of ROS and pollen abortion. MT-1-4b, encodes a type 1 small Cys-rich and metal binding protein, has •OH and O_2_•^−^ scavenging activity. Reducing its expression causes increased ROS accumulation and decreased pollen fertility in rice (*Oryza sativa*) [18].

Protecting and stabilizing proteins play a critical role in heat shock response in plants. Heat shock proteins (HSPs) which perform chaperone function by refolding damaged proteins and preventing the formation of aggregates, are one of the most important gene families for plants to survive during high temperature stress [19]. Frank et al. [17] found that numerous small HSPs, *HSP70* and *HSP90*, could have higher basal expression levels in the heat-tolerant tomato cultivar rather than the heat-sensitive cultivar. Similarly, heterogeneous over-expression of *Arabidopsis HSP101* in tobacco (*Nicotiana tabucum*) and cotton (*Gossypium hirsutum*) resulted in higher pollen thermotolerance and seed production [20]. Besides protein refolding, heat-induced protein should be degraded by 26S proteasome and autophagy in plants [21,22]. The mutant of E3 ligase the carboxyl terminus of the Hsc70-interacting protein (CHIP) mutant exhibited higher temperature sensitivity and accumulated more heat-induced ubiquitinated insoluble proteins in *Arabidopsis* leaves [21]. Most autophagy-related genes (*ATGs*) mutants exhibited heat-sensitivity and defects in anther development and pollen germination [23,24,25]. However, the regulatory network and thermotolerance mechanisms of protein protection in anther and pollen development process remain to be determined.

Melatonin is a pleiotropic signal molecule that regulates diverse growth and development processes and provides physiological protections against various environmental stresses [26,27]. Exogenous application or endogenous manipulation of melatonin enhanced plant tolerance to both abiotic and biotic stresses [15,28,29,30,31,32]. The excellent properties of melatonin as an anti-oxidative molecule, an ROS scavenger and a regulator of anti-oxidative system have been widely demonstrated in plants [26,33]. Recently, several studies reported that melatonin promoted the protection and degradation of denatured proteins by inducing the transcript levels of HSPs and the formation of autophagy in response to abiotic stresses [15,30,34]. With a similar structure and common biosynthetic pathway to phytohormone auxin/indole-3-acetic acid (AUX/IAA), melatonin also promotes shoot and root growth in several species such as *Arabidopsis* [35], Bermuda grass (*Cynodon dactylon*) [36], rice [37] and soybean (*Glycine max*) [38]. Meanwhile, recent studies have demonstrated that melatonin regulates reproduction in plants. Exogenous melatonin enhanced seed number, production and fatty acid content in soybean [38]. Over-expression of serotonin *N*-acetyltransferase (*SNAT*) gene exhibited increased panicle number and grain yield in rice [39]. However, the exact role of melatonin during plant reproduction is still not completely understood.

How high temperature-induced pollen abortion is regulated in plants remains a critical unrequited query. We hypothesized that melatonin might play a regulatory role in pollen thermotolerance in tomato. In this study, we have found that exogenous melatonin increases pollen viability and germination under high temperature. Moreover, melatonin alleviates ROS production and induces activity of antioxidant enzymes. Finally, we show that melatonin protects degradation of tapetum cells and pollen by promoting the higher levels of HSPs and ATGs transcripts and autophagic signaling in tomato anthers. This study deepens the understanding of melatonin functions in plant reproductive development and may have important implications for breeding heat-tolerant crops.

## 2. Results and Discussion

### 2.1. Melatonin Alleviates Pollen Abortion under High Temperature

**Figure 1:**
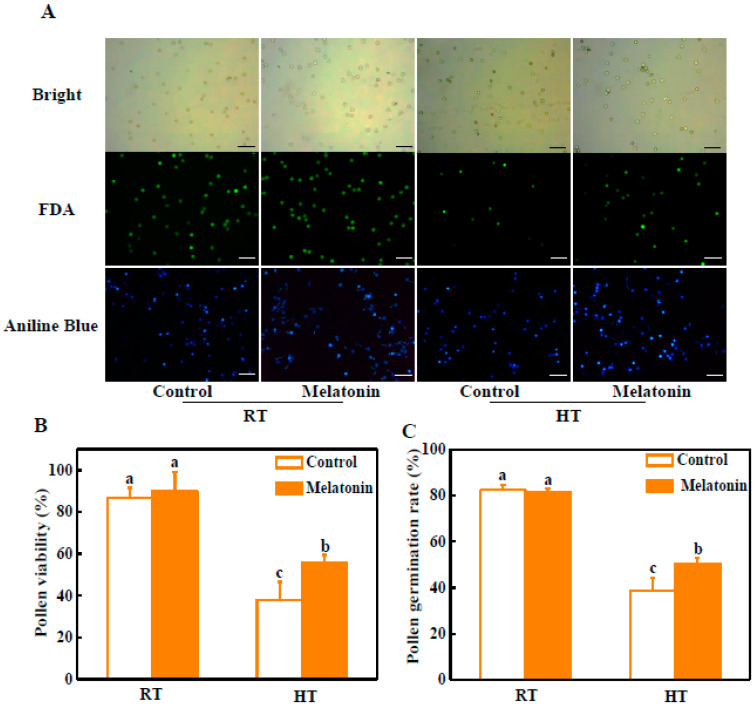
Melatonin alleviates the decrease of pollen viability and pollen germination after 3 h high temperature stress. (**A**) Detection of pollen viability by fluorescein diacetate (FDA) staining and assay of pollen germination by Aniline Blue staining in vitro. Bars = 200 μm; (**B**) Statistical pollen viability in (**A**). At least 500 pollens for each treatment were used for the quantification; (**C**) Statistical pollen germination rate in (**A**). At least 500 pollens for each treatment were used for the quantification. The data shown are the average of four replicates, with the standard errors shown by vertical bars. Means denoted by the same letter did not significantly differ at *p* < 0.05, according to Tukey’s test.

At normal temperature (at 25 °C), viable pollen grains were dominant by fluorescein diacetate (FDA) staining, with 13.4% aborted grains (Figure 1A,B). Pollen viability was slightly increased with melatonin pre-treatment. Compared to control pollen grains, the most of pollen grains was aborted, only with 24.8% viable grains after 3 h high temperature treatment (42 °C). Importantly, melatonin pre-treatment greatly alleviated high temperature-induced pollen abortion. The pollen viability was increased by 45.7% compared with untreated plants under high temperature (Figure 1A,B). Similarly, the mean germination ratio of pollen grains with melatonin pretreatment was significantly higher than that of control under high temperature (Figure 1A,C). Only 38.7% pollens of control germinated compared with 50.5% pollens of melatonin-pretreatment in vitro after 3 h high temperature treatment (42 °C) (Figure 1C).

Pollen grain viability is one of the most decisive factors controlling crop yield. However, pollen grains are more sensitive to high temperature than ovules within the florets [40]. In this study, we found that the pollen viability and germination of melatonin-treated plants were increased under high temperature (Figure 1). Previous study reported that melatonin enhanced seed’s number and yield of soybean plants under field conditions [38]. These findings indicate that melatonin probably regulates the development of pollen and the maintaining of microspore activity to increase seed’s number during plant reproductive growth phase.

### 2.2. Melatonin Enhances ROS Scavenging and Antioxidant Enzyme Activities under High Temperature

**Figure 2:**
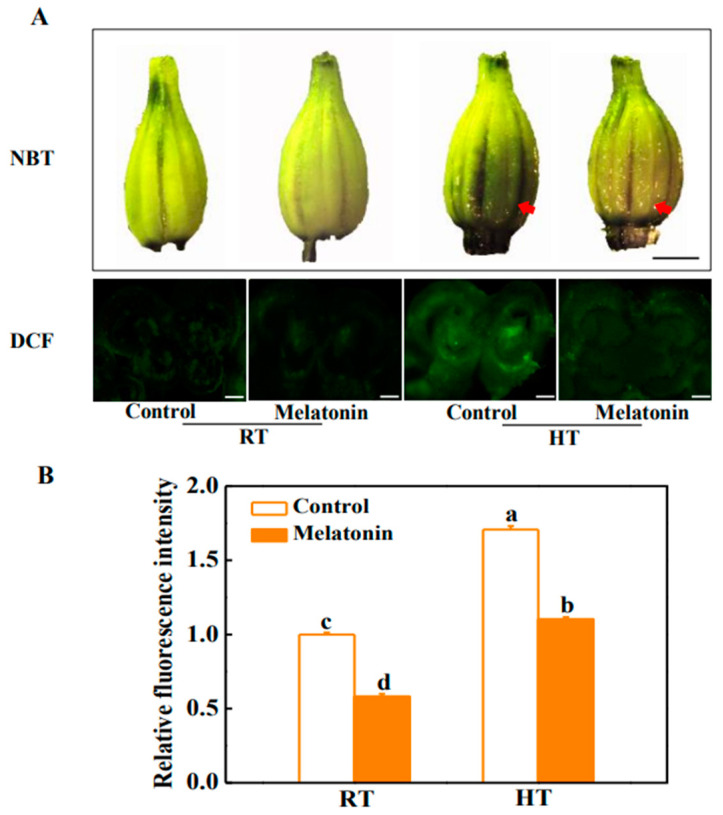
Melatonin enhances the ability of scavenging reactive oxygen species (ROS) in tomato anthers under high temperature stress. (**A**) Detection of superoxide anion (O_2_•^−^) by nitro blue tetrazolium (NBT) staining and assay of hydrogen peroxide (H_2_O_2_) by 2,7-dichlorofluorescein diacetate (DCF) staining. Bars = 1.5 mm (upper panel) and bars = 60 μm (lower panel). In the upper panel, the red arrow indicates the darker of the anther color, and the deeper of the tissue slice fluorescence, the more ROS has accumulated, which means that melatonin efficiently removed the ROS and alleviated ROS production in anthers under high temperature. (**B**) Relative fluorescence intensity is based on the DCF staining. The data shown are the average of four replicates, with the standard errors shown by vertical bars. Means denoted by the same letter did not significantly differ at *p* < 0.05, according to Tukey’s test.

Melatonin has also been reported as an efficient modulator under high temperature by enhancing activities of antioxidant enzymes and scavenging ROS in plants [41]. By using nitro blue tetrazolium (NBT) as a probe for superoxide anion (O_2_•^−^), we found that ROS accumulation was induced by high temperature in the anthers after 3 h high temperature treatment (42 °C) (Figure 2A). In addition, staining the anthers with 2,7-dichlorofluorescein diacetate (DCF) further confirmed that the anthers of heat-treated plants accumulated more H_2_O_2_ than plants under normal condition (Figure 2). Moreover, melatonin effectively alleviated H_2_O_2_ accumulation in anthers under high temperature. The H_2_O_2_ content was decreased by 35.3% in melatonin-pretreated plants compared with that in untreated plants after 3 h high temperature stress (Figure 2B). Further analysis of the tissular localization of H_2_O_2_ revealed that H_2_O_2_ accumulation occurred primarily in the tapetum cells of tomato anthers under high temperature, whilst heat-induced H_2_O_2_ accumulation of tapetum cells was much arrested in melatonin pretreated plants (Figure 2).

**Figure 3:**
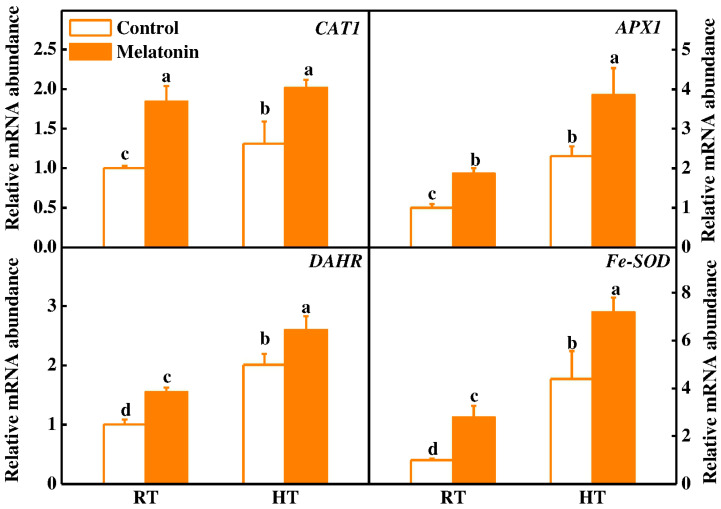
Expression of antioxidant genes in tomato anthers after 3 h high temperature stress. The data shown are the average of four replicates, with the standard errors shown by vertical bars. Means denoted by the same letter did not significantly differ at *p* < 0.05, according to Tukey’s test.

**Figure 4:**
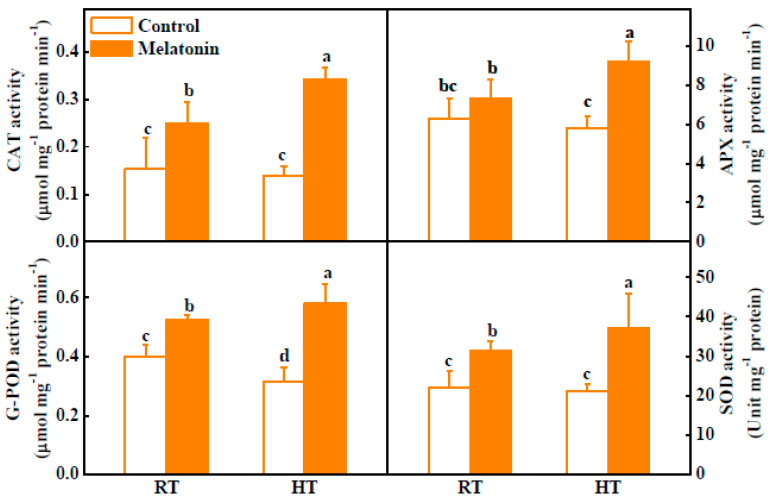
Activities of CAT, APX, G-POD and SOD in tomato anthers after 3 h high temperature stress. The data shown are the average of four replicates, with the standard errors shown by vertical bars. Means denoted by the same letter did not significantly differ at *p* < 0.05, according to Tukey’s test.

Melatonin can directly detoxify ROS and indirectly stimulate antioxidant enzymes to scavenge ROS in plant exposure to stress [42]. To investigate the role of melatonin in the regulation of the plant antioxidant system, qRT-PCR was used to analyze the gene expression of *CAT1*, *APX1*, *DAHR* and *Fe-SOD*. The transcript levels of four antioxidant-related genes increased by 55.1–172.7% after melatonin pretreatment under normal condition (Figure 3). Meanwhile, high temperature induced expression of all four genes under high temperature, and melatonin pretreatment further increased expression of these antioxidant enzymes (Figure 3). Consistent with the changes in gene transcription, the activities of catalase (CAT), ascorbate peroxidase (APX), guaiacol peroxidase (G-POD) and superoxide dismutase (SOD) were significantly increased by 146.0%, 58.8%, 83.5% and 74.5% in melatonin-pretreated anthers compared with untreated plants under high temperature, respectively (Figure 4). These findings clearly highlight the active participation of melatonin in alleviating high temperature-induced ROS in tomato anthers. Interestingly, the protective role of melatonin was also observed in other plant species challenged with different abiotic stresses [28,32,43]. In summary, we have demonstrated that pretreatment with melatonin significantly alleviated ROS in tomato anthers challenged with high-temperature stress. This relief could possibly be inter-related with pretreatment of melatonin, which further stimulated its endogenous thereby acting as an antioxidant, direct scavenger of ROS or indirectly by stimulating the expression of anti-oxidative enzymes [42].

### 2.3. Melatonin Protects Stability of Tapetum Cells by Inducing Expression of HSP and ATG Genes and Formation of Autophagosomes under High Temperature

**Figure 5:**
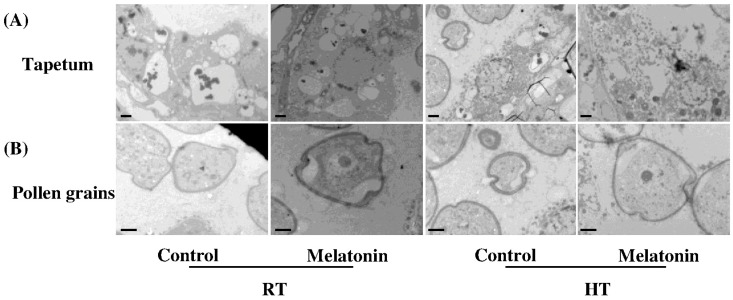
Ultrastructures of: tapetum cells (**A**); and pollen grains (**B**) at the early uninuclear microspore stage in tomato. Pollen is indicated with black arrows. Bars = 2 μm.

High temperatures significantly alter reproductive functioning in plants by distorting the development and functioning of the male gametophyte followed by compromised fruit yield [44,45]. The development of pollen is nurtured by sporophytic cell layer, known as tapetum [46]. Therefore, it becomes obvious that any developmental defects in the pollen induced by high temperature are often seen in the tapetum [47,48]. In this study, we found that the tapetum cells were well organized, exhibiting appropriate distribution and organization of the organelles under control conditions by transmission electron micrograph (Figure 5). Whist, high temperature caused the significant degeneration of tapetum cells and pollen deformity with degenerated nuclei at the early uninuclear microspore stage (Figure 5). Importantly, pretreatment with melatonin significantly restores high temperature-induced degeneration of tapetum cells and pollen deformity (Figure 5). Tapetum cells which provide nutrients to pollen grains are sensitive to high temperature, and their degeneration time was modified under high temperature stress [49,50]. Furthermore, transcriptome analysis carried in rice have already confirmed the sensitivity of the tapetum towards high temperature stress [51]. Hence, melatonin protects stability of tapetum cells and avoids pollen deformity under high temperature stress.

**Figure 6:**
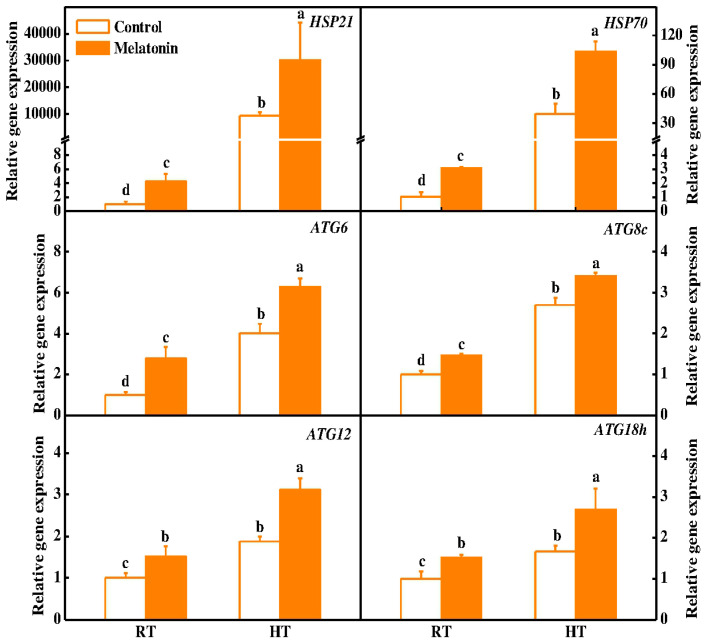
Expression of *HSP* and *ATG* genes in tomato anthers after 3 h high temperature stress. The data shown are the average of four replicates, with the standard errors shown by vertical bars. Means denoted by the same letter did not significantly differ at *p* < 0.05, according to Tukey’s test.

To neutralize high temperature brought ill effects, plants trigger the expression of heat shock protein (HSP) chaperones [52]. Recent studies have shown the active involvement of melatonin in up-regulating the expression of *HSP* family genes and protecting protein stability under high-temperature stress [15,30]. However, the co-relation between melatonin and HSPs in regulating tapetum restoration under heat stress is poorly understood. In tomato plants, ameliorative roles of small HSPs and *HSP70* have been reported in the early stages of pollen developmental, signifying their roles in the cells during environmental stresses [53,54,55]. Interestingly, we found that pretreatment with melatonin significantly induces the expression of *HSP21* (3.23 folds) and *HSP70* (2.6 folds) in tomato anthers upon exposure to high-temperature stress when compared to control ones (Figure 6). Furthermore, small HSPs have also been shown to play significant roles in regulating the cell membrane integrity [56]. Therefore, it can be concluded that melatonin induces the expression of *HSP21* and *HSP70*, which eventually restore the stability of tapetum upon exposure to high-temperature stress as observed in TEM micrographs (Figure 5).

**Figure 7:**
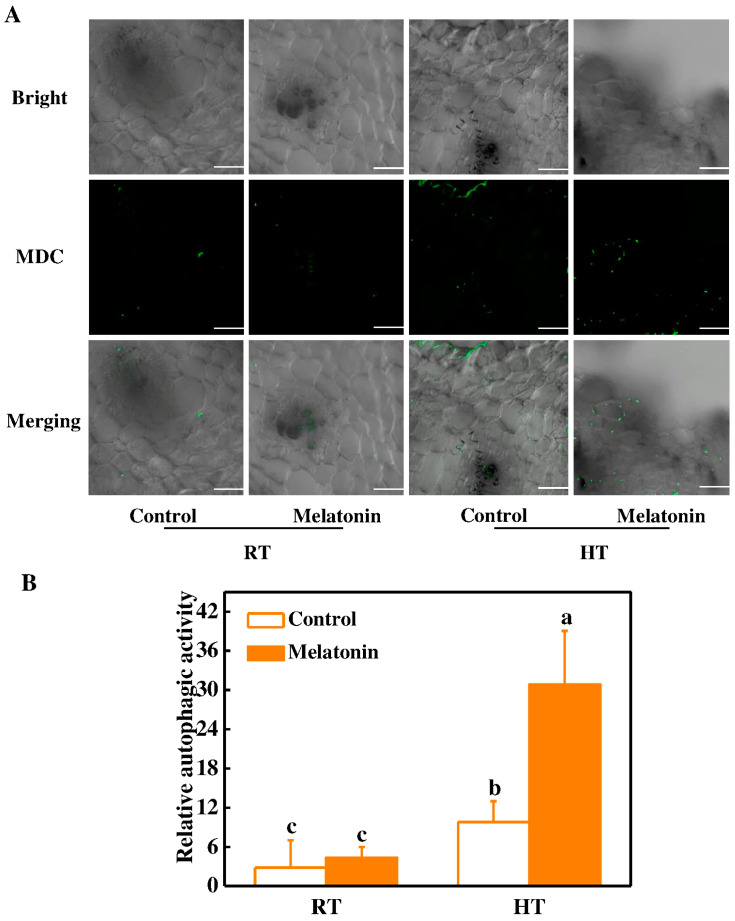
Visualization of the accumulation of autophagosomes in tomato anthers after 3 h high temperature stress. (**A**) MDC-stained autophagosomes in the anthers. The anthers were stained with MDC and visualized by confocal microscopy. MDC-stained autophagosomes were shown as green signals, just as indicated with white arrows. Bars = 25 μm; (**B**) Relative autophagic activity normalized to the activity of the control plants in (**A**). The MDC-stained autophagosomes in the anthers at each treatment were quantified to calculate the autophagic activity relative to control plants, which was set to 1. More than 300 mesophyll cells for each treatment were used for the quantification. Mel, melatonin pretreatment. The data shown are the average of four replicates, with the standard errors shown by vertical bars. Means denoted by the same letter did not significantly differ at *p* < 0.05, according to Tukey’s test.

Autophagy is a conserved eukaryotic pathway for degradation of cellular components and plays critical roles in plant growth and development [57,58]. Autophagy-related gene (*ATG*) mutants exhibited sterile phenotype due to defective pollen maturation and limited anther in rice [24]. Meanwhile, we recently reported that melatonin induces the expression of *ATG* genes and formation of autophagy in response to heat stress [15]. To further seek advances on the alleviatory roles of melatonin in the restoration of tapetum, we investigated the expression of several *ATG* genes (*ATG6*, *ATG8c*, *ATG12* and *ATG18h*). It was observed that the expression of these *ATG* genes was induced in anthers by melatonin pretreatment under normal conditions (Figure 6). Importantly, the expression of *ATGs* was further increased by 26.0–64.7% in melatonin-pretreated anthers compared with control plant anthers after 3 h high temperature stress (Figure 6). In addition, we used a fluorescent dye monodansylcadaverine (MDC) to detect autophagic activity in anthers. In the control plants, few MDC-stained autophagosomes were observed (Figure 7A). In contrast, numerous MDC-stained autophagosomes were detected in anthers after 3 h high temperature stress. Pretreatment with melatonin further stimulates the formation of autophagosomes for degrading heat-induced misfolded or degraded proteins in anthers (Figure 7). Recently, many studies have indicated that autophagy increases plant stress tolerance by degrading and recycling of cytoplasmic damaged proteins and organelles, and quenching of ROS accumulation in plants [14,23,59]. Interestingly, melatonin was reported to induce *ATG* gene expression and autophagy formation in various plants [15,34,60]. Taken together, our results provided comprehensive evidence on the protective roles of melatonin in restoring stability of tapetum cells by inducing of *HSP* and *ATG* genes and formation of autophagy upon exposure to high temperature stress.

## 3. Materials and Methods

### 3.1. Plant Materials, Growth Conditions and Experimental Design

Tomato (*Solanum lycopersicum* L. cv. Micro-Tom) seeds were germinated in a growth medium filled with a mixture of vermiculite and perlite (7:1 = *v*:*v*) within a growth chamber. When the fourth true leaves were fully expanded, the seedlings were transferred into plastic pots (40 cm × 25 cm × 15 cm) filled with Hoagland’s nutrient solution for hydroponic culture. The growth conditions were as follows: a photoperiod of 14 h day and 10 h night, a temperature of 25/23 °C (day/night), a mean relative humidity of 80%, and a photosynthetic photon flux density (PPFD) of 600 μmol·m^−2^·s^−1^. To analyze the melatonin-induced pollen thermotolerance, the roots of tomato seedlings were pretreated with 20 µM melatonin, when the first inflorescence of tomato plants were fully developed, and the other inflorescences were budded. The melatonin treatment was given for 7 days, followed by 42 °C high temperature for 3 h in a growth chamber. The samples of stamen or pollen were collected for detection of the physiological, biochemical and molecular indexes. Four replicates were used for each treatment, and each replicate with consisted of 12 plants.

### 3.2. Identification of Pollen Viability and Germination, and Abnormal Pollen Grains

The pollen viability in tomatoes was detected by fluorescein diacetate (FDA) (Aladdin, F109384) as previously described [61]. FDA was dissolved in acetone and stored as a stock solution (2 mg·mL^−1^), and a drop of the 0.01% stock solution (diluted in 0.5 M sucrose solution) was placed on the slide to stain pollen grains. The viable pollen grains with fluorescence was determined by fluorescence microscope (Leica, Wetzlar, Germany). For in vitro pollen germination assay, pollen was sprinkled in a growth medium containing 0.1% agar, 0.29 M sucrose, 1.27 mM Ca(NO_3_)_2_, 0.16 mM H_3_BO_3_, 0.8 mM MgSO_4_, and 1 mM KNO_3_ (pH 6.5) [62]. The pollen tube grew within 1.5 h at room temperature in the dark, and then staining by 0.1% aniline blue (B8563, Sigma-Aldrich, St. Louis, MO, USA). Pollen germination percentages were measured by fluorescence microscope (DFC425C, Leica, Wetzlar, Germany).

### 3.3. Histochemical Staining of ROS

For O_2_•^−^ detection, anthers were infiltrated with 0.1 mg·mL^−1^ nitro blue tetrazolium (NBT) in 25 mM K-HEPES buffer (pH 7.8) and incubated at 25 °C in the dark for 1 h. After 95 °C boiled in 95% (*v*/*v*) ethanol for 15–20 min, take photographs with the anthers as described [63]. For ROS detection, transverse sections of anthers were infiltrated with 50 mM PBS (pH 7.4) for 30 min and later stained with fluorescent dye 2,7-dichlorofluorescein diacetate (DCF) (35848, Sigma-Aldrich, St. Louis, MO, USA) for 10 min. The sections were washed by PBS buffer for three times, and afterwards the ROS fluorescent signaling were detected by a Leica DM4000B microscope equipped with a Leica DFC425C camera [64].

### 3.4. Antioxidant Enzyme Extraction and Activity Assays

For enzyme extraction, 0.1 g of fresh stamens was refrigeration-rubbed by Freezer Mixer (TissueLyser II, Retsch, Germany), then added to 800 μL ice-cold 50 mM PBS (pH 7.8) containing 0.2 mM EDTA and 2% polyvinylpyrrolidone (*w*/*v*) and homogenized fully by vortex. The homogenate was centrifuged at 12,000× *g* for 20 min at 4 °C, and the supernatant was collected. The protein content was determined using Bradford’s method [65] and this supernatant was used to measure the activities of superoxide dismutase (SOD), catalase (CAT), ascorbate peroxidase (APX) and guaiacol peroxidase (G-POD) enzymes. Specifically, SOD activity was determined by measuring its ability to inhibit the photochemical reduction of NBT as described [66]. CAT activity was determined by following the consumption of H_2_O_2_ at 240 nm [67]. The reaction mixture contained 25 mM PBS (pH 7.0), 10 mM H_2_O_2_ and enzyme extract. APX activity was measured through the decrease in absorbance at 290 nm [68]. The reaction mixture contained 25 mM PBS (pH 7.0), 0.25 mM ascorbate (AsA), 1 mM H_2_O_2_ and enzyme extract. G-POD activity was assessed using guaiacol as a substrate and was determined at 470 nm with some modifications [69] The reaction mixture contained 25 mM PBS (pH 7.0), 0.05% guaiacol, 1 mM H_2_O_2_ and enzyme extract.

### 3.5. Total RNA Extraction and Gene Expression Analysis

Total RNA was isolated from tomato stamens using RNA simple total RNA kit (DP419, Tiangen, Shanghai, China) according to the manufacturer’s instructions. One microgram of total RNA was used to reverse transcribed for cDNA template using the HiScript^®^ II Q RT SuperMix for qPCR (Vazyme, Nanjing, China, R223-01). The qRT-PCR analysis was performed using the SYBR Green PCR Master Mix (Takara, Tokyo, Japan) on the AB StepOnePlus™ Real-Time PCR System (Applied Biosystems, Foster City, CA, USA) under default thermal cycling conditions with an added melt curve. PCR program included pre-denaturation at 95 °C for 3 min, followed by 40 cycles of denaturation at 95 °C for 30 s, annealing at 58 °C for 20 s, and extension at 72 °C for 30 s. Relative transcript level was calculated according to the method as described [70]. The Gene-specific primers for qRT-PCR were designed based on their cDNA sequences (Table S1), which contains the corresponding genes, such as *ATGs*, *HSPs*, *CAT1*, *cAPX1*, *DHAR*, and *Fe-SOD* genes, and the marker gene *actin* that was used as an internal control.

### 3.6. Transmission Electron Microscopy (TEM)

For observation of sub-cellular structures, anthers of different treatments at the early uninuclear microspore stage were first fixed with 2.5% glutaraldehyde in PBS (0.1 M, pH 7.0) for 24 h at 4 °C; washed three times in the PBS (0.1 M, pH 7.0) for 15 min at each step; and then post-fixed with 1% osmium tetraoxide for 1–2 h and washed three times in the PBS (0.1 M, pH 7.0) for 15 min at each step. Secondly, anthers were dehydrated by a graded series of ethanol (30%, 50%, 70%, 80%, 90%, 95% and 100%; *v*/*v*) for about 15–20 min at each step, and then transferred to absolute acetone for 20 min. Thirdly, anthers were placed at 1:1 mixture of absolute acetone and the final spur resin mixture for 1 h at room temperature, then transferred to 1:3 mixture of absolute acetone and the final resin mixture for 3 h and to final spur resin mixture for overnight. Lastly, anthers were placed in eppendorf contained spur resin and heated at 70 °C for more than 9 h. The anther’s ultra-thin sections (70 nm) were prepared on an ultramicrotome (Leica EM UC7, Wetzlar, Germany) with a diamond knife and stained with 2% uranyl acetate on grids followed by a lead citrate staining solution for 5–10 min respectively and examined with Hitachi Model H-7650 TEM (Hitachi, Tokyo, Japan) at an accelerating voltage of 75 kV to observe the sstability of tapetum cell [71,72].

### 3.7. Observation of Autophagic Activity Using MDC Staining

To visualize the autophagosomes, mature tomato stamens were infiltrated with 100 μM of mondansylcadaverine (MDC) (Sigma-Aldrich, 30,432) for 30 min, followed by rinsing with PBS (P1020, Solarbio, Beijing, China) and cutting into sections. Fluorescence were observed with the A1-SHS confocal microscope (Nikon, Tokyo, Japan) as previously described [14].

### 3.8. Statistical Analysis

The data were analyzed using ANOVA and expressed as the mean value for each treatment. A Turkey’s test (*p* < 0.05) was performed to evaluate the treatment effects. At least four independent replicates were conducted for each determination.

## 4. Conclusions

In conclusion, our results demonstrate that melatonin as a signaling molecule has multiple protective functions for pollen viability under high temperature. First, melatonin alleviates high temperature-induced inactivation of pollen and inhibition of pollen germination. Second, melatonin decreases ROS accumulation in the anthers by inducing activities of antioxidant enzymes. Third, melatonin is also important for the stability of organelles in the tapetum. Our work provides new insights into melatonin-regulated reproductive development and stress tolerance mechanism in plants.

## Supplementary Materials

The following is available online, Table S1: Primers used for qRT-PCR assays in tomato anthers.
