# Correction: Qi et al. Melatonin Alleviates High Temperature-Induced Pollen Abortion in *Solanum lycopersicum*. *Molecules* 2018, *23*, 386

**DOI:** 10.3390/molecules29153641

**Published:** 2024-08-01

**Authors:** Zhen-Yu Qi, Kai-Xin Wang, Meng-Yu Yan, Mukesh Kumar Kanwar, Dao-Yi Li, Leonard Wijaya, Mohammed Nasser Alyemeni, Parvaiz Ahmad, Jie Zhou

**Affiliations:** 1 Department of Horticulture/Zhejiang Provincial Key Laboratory of Horticultural Plant Integrative Biology, Zhejiang University, Hangzhou 310058, China; qizhenyu@zju.edu.cn (Z.-Y.Q.); keisha@zju.edu.cn (K.-X.W.); 11416057@zju.edu.cn (M.-Y.Y.); kanwar@zju.edu.cn (M.K.K.); 2 Agricultural Experiment Station, Zhejiang University, Hangzhou 310058, China; 3 Chinese Academy of Agricultural Mechanization Sciences, Beijing 10083, China; daoyili@126.com; 4 Department of Botany and Microbiology, Faculty of Science, King Saud University, Riyadh 11451, Saudi Arabia; lwijaya@ksu.edu.sa (L.W.); mnyemeni@ksu.edu.sa (M.N.A.); pganaie@ksu.edu.sa (P.A.)

In the original publication [1], there was a mistake in Figure 2A as published. The representative DCF fluorescence image of Control under room temperature (2nd row, panel 1) was overwritten by a redundant image. The corrected Figure 2 appears below. The authors state that the scientific conclusions are unaffected. This correction was approved by the Academic Editor. The original publication has also been updated.

## Figures and Tables

**Figure 2:**
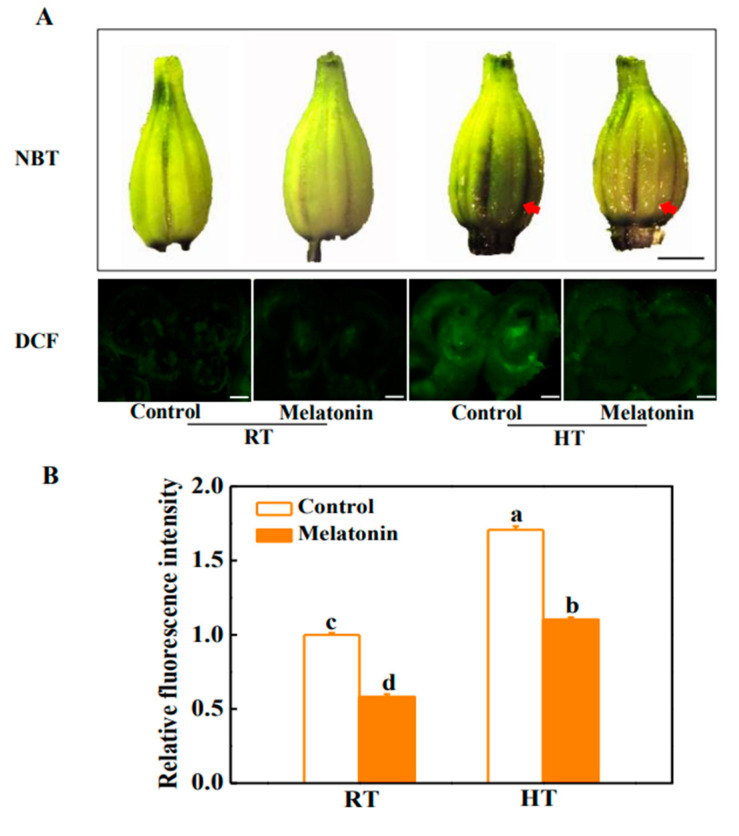
Melatonin enhances the ability of scavenging reactive oxygen species (ROS) in tomato anthers under high temperature stress. (**A**) Detection of superoxide anion (O_2_•^−^) by nitro blue tetrazolium (NBT) staining and assay of hydrogen peroxide (H_2_O_2_) by 2,7-dichlorofluorescein diacetate (DCF) staining. Bars = 1.5 mm (upper panel) and bars = 60 μm (lower panel). In the upper panel, the red arrow indicates the darker of the anther color, and the deeper of the tissue slice fluorescence, the more ROS has accumulated, which means that melatonin efficiently removed the ROS and alleviated ROS production in anthers under high temperature. (**B**) Relative fluorescence intensity is based on the DCF staining. The data shown are the average of four replicates, with the standard errors shown by vertical bars. Means denoted by the same letter did not significantly differ at *p* < 0.05, according to Tukey’s test.
